# A Case of Enfortumab Vedotin-Associated Diabetic Ketoacidosis With Severe Insulin Resistance in a Nondiabetic Woman

**DOI:** 10.1210/jcemcr/luae212

**Published:** 2024-11-25

**Authors:** Rachel Hovelroud, Sarah Goh Xiu Ming, Donald S A McLeod, Peter J Donovan, Gary Ng, Maree Mungomery

**Affiliations:** Department of Diabetes and Endocrinology, Townsville University Hospital, Douglas QLD 4814, Australia; Department of Diabetes and Endocrinology, Royal Brisbane & Women's Hospital, Herston QLD 4006, Australia; Department of Diabetes and Endocrinology, Royal Brisbane & Women's Hospital, Herston QLD 4006, Australia; Department of Diabetes and Endocrinology, Royal Brisbane & Women's Hospital, Herston QLD 4006, Australia; Department of Medical Oncology, Royal Brisbane & Women's Hospital, Herston QLD 4006, Australia; Department of Diabetes and Endocrinology, Royal Brisbane & Women's Hospital, Herston QLD 4006, Australia

**Keywords:** enfortumab vedotin, diabetic ketoacidosis, insulin resistance, urothelial carcinoma, antibody-drug conjugate, monomethyl auristatin E

## Abstract

Enfortumab vedotin is a novel antibody-drug conjugate (ADC) approved to treat urothelial carcinoma. One rarely reported adverse effect has been life-threatening diabetic ketoacidosis (DKA) driven by profound insulin resistance. We report a case of a 62-year-old nondiabetic woman with metastatic urothelial carcinoma who experienced DKA following her third dose of enfortumab vedotin, with extreme insulin requirements of > 1000 units daily, and full resolution of insulin requirement by day 7 of admission. Including this case, 3 of 9 reported patients with enfortumab vedotin–associated DKA have survived. Monomethyl auristatin E (MMAE), the cytotoxic component of enfortumab vedotin, is the likely cause, although the exact mechanism remains unclear. This rare clinical event challenges the usual protocols and practice surrounding insulin infusion administration, and this case provides evidence to assist in understanding the mechanism by which enfortumab vedotin causes ketoacidosis.

## Introduction

Locally advanced or unresectable urothelial carcinoma is an aggressive and incurable disease with a median overall survival of less than 2 years with platinum-based chemotherapy and immune checkpoint inhibitor therapy [1]. Enfortumab vedotin is an antibody-drug conjugate (ADC) that confers prolonged survival in patients with previously treated disease and when used in combination with pembrolizumab [2, 3].

Enfortumab vedotin has 3 different components. *Enfortumab* is a human monoclonal antibody specific to transmembrane protein nectin-4, which is overexpressed in urothelial carcinoma. This antibody is linked to a payload through a protease sensitive valine-citrulline dipeptide linker. The cytotoxic payload *vedotin* is a microtubule disrupting agent, *monomethyl auristatin E* (MMAE). Once enfortumab vedotin is internalized, MMAE is released through proteolytic cleavage resulting in intracellular release of MMAE and apoptosis of tumor cells [4, 5].

Hyperglycemia was a relatively common treatment-related adverse event (TRAE) of enfortumab vedotin, occurring in 6.8% of patients in the phase 3 EV-301 study, even in patients without prior diabetes [6]. Diabetic ketoacidosis (DKA) is a rare complication of enfortumab vedotin therapy associated with high mortality. In the phase 1 EV-101 trial, there were 4 fatal TRAEs, including 1 from DKA [7]. Subsequently there have been 8 case reports of enfortumab vedotin–associated DKA described in the literature, with 2 patients surviving to discharge [8‐16]. This case represents the third patient to have survived, with further evidence supporting the presence of transient reversible severe insulin resistance, and it further clarifies the timeline of this TRAE and its management.

## Case Presentation

A 62-year-old woman presented to hospital with lethargy, anorexia, nausea, fever, and rash 7 days after her third dose (Cycle 1, Day 15) of enfortumab vedotin (1.25 mg/kg; weight 65.5 kg). She had received cisplatin-gemcitabine chemotherapy 2 years prior, and subsequently pembrolizumab immune checkpoint inhibitor therapy, with the last dose given 5 weeks before presentation. Enfortumab vedotin was commenced as third-line treatment after imaging showed progressive metastatic disease. Her other comorbidities included hypertension, dyslipidemia, and a body mass index of 25.6 kg/m^2^. She had no known history of diabetes, with normal blood glucose level of 6.9 mmol/L (124 mg/dL) (normal reference range: 3.0-7.8 mmol/L; 54-140 mg/dL) 1 week earlier.

## Diagnostic Assessment

At admission, she was found to be in DKA, as supported by an elevated blood glucose level of 22.0 mmol/L (396 mg/dL) (normal reference range: 3.0-7.8 mmol/L; 54-140 mg/dL) and elevated capillary ß-hydroxybutyrate of 5.8 mmol/L (33.7 mg/dL) (normal reference range: < 0.6 mmol/L; < 3.49 mg/dL) measured using the Freestyle Libre Optimum Neo blood glucose and ketone meter, as well as a high anion gap metabolic acidosis (Table 1). She was also febrile to 38.1 °C and stable hemodynamically. Neutropenia was present but septic screen did not reveal an infective source.

**Table 1.** Laboratory data on admission

Blood test	Result	Reference range
pH (venous blood gas)	**7.21**	7.32-7.43
Bicarbonate	**12 mmol/L (12 mEq/L)**	22-32 mmol/L (22-32 mEq/L)
Anion gap	**18 mmol/L (18 mEq/L)**	4-13 mmol/L (4-13 mEq/L)
Lactate	**2.8 mmol/L (25.2 mg/dL)**	0.5-2.2 mmol/L (4.5-19.8 mg/dL)
pCO2 (venous blood gas)	**26 mmHg**	38-54 mmHg
Creatinine	**94 µmol/L (1.06 mg/dL)**	36-73 µmol/L (0.41-0.83 mg/dL)
Albumin	36 g/L (3.6 g/dL)	35-50 g/L (3.5-5.0 g/dL)
Glucose	**22.0 mmol/L (396 mg/dL)**	3.0-7.8 mmol/L (54-140 mg/dL)
Sodium	135 mmol/L (135 mEq/L)	135-145 mmol/L (135-145 mEq/L)
Potassium	4.1 mmol/L (4.1 mEq/L)	3.5-5.2 mmol/L (3.5-5.2 mEq/L)
Calcium	2.60 mmol/L (10.4 mg/dL)	2.10-2.60 mmol/L (8.4-10.4 mg/dL)
Phosphate	**0.49 mmol/L (1.52 mg/dL)**	0.75-1.50 mmol/L (2.3-4.6 mg/dL)
Magnesium	0.80 mmol/L (1.94 mg/dL)	0.70 -1.10 mmol/L (1.7-2.7 mg/dL)
Capillary ketone level	**5.8 mmol/L (33.7 mg/dL)**	<0.6 mmol/L (<3.49 mg/dL)
C-peptide level (fasting)	**6.3 nmol/L (19.0 ng/mL)**	0.3-1.4 nmol/L (0.91-4.23 ng/mL)

Abnormal values are shown in bold. Values given are in International System of Units (SI). Values in parentheses are conventional units.

The corresponding C-peptide level on admission was elevated at 6.3 nmol/L (19.0 ng/mL) (fasting reference range: 0.3-1.4 nmol/L; 0.91-4.23 ng/mL). Her glycated hemoglobin (HbA1c) level was 6.7% (50 mmol/mol) (normal reference range: < 5.7%; < 39 mmol/mol) with negative anti-glutamic acid decarboxylase, anti-islet antigen 2, and anti-insulin antibodies (Table 1).

## Treatment

Our patient was commenced on an intravenous insulin infusion, initially at 3 units/hour, along with intravenous 0.9% sodium chloride at 500 mL/hour. She was given filgrastim and intravenous piperacillin-tazobactam and vancomycin for febrile neutropenia.

**Figure 1.**
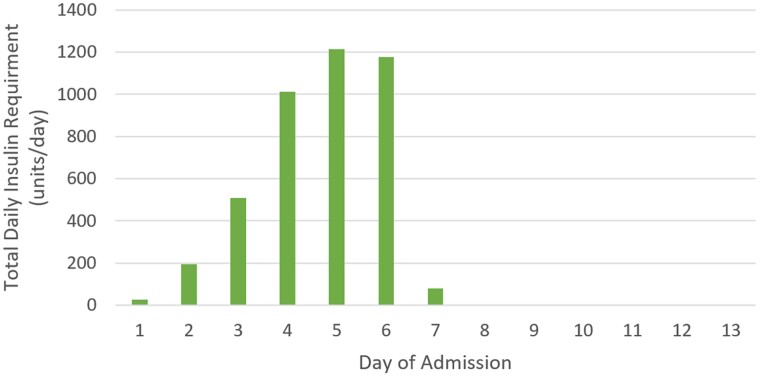
Total daily insulin requirement (sum of daily intravenous and subcutaneous insulin for each day during admission).

Hyperglycemia, ketosis, and metabolic acidosis remained refractory to insulin therapy, and the intravenous insulin infusion was uptitrated (Table 2, Fig. 1), with the addition of basal-bolus subcutaneous insulin on day 2 (comprising 40 units of insulin glargine daily and 16 units of insulin aspart 3 times a day). Maintenance intravenous 0.9% sodium chloride at 100 mL/hour was administered with regular clinical assessment of fluid status. The intravenous insulin infusion rate peaked at 50 units/hour by day 5, with a peak total daily insulin dose of 1213 units. At this time, ketoacidosis persisted, and blood glucose remained elevated (Table 2, Fig. 1); however, clinically, our patient remained stable with no organ failure. As 25 units/hour represented the maximum programmable dose of our intensive care-level infusion pump, we achieved 50 units/hour by doubling the standard concentration of insulin in the driver syringe. We monitored closely for hyperglycemia resolution as previous case reports suggested that reversal of insulin resistance could rapidly occur.

**Table 2.** Biochemical values during admission

Day of admission	1	2	3	4	5	6	7	8	9	10	11	12
**Daily peak glucose level (reference range: 3.0-7.8 mmol/L [54-140 mg/dL])**	20.3 mmol/L (366 mg/dL)	17.9 mmol/L (323 mg/dL)	18.5 mmol/L (333 mg/dL)	18.2 mmol/L (328 mg/dL)	14.2 mmol/L (256 mg/dL)	12.7 mmol/L (256 mg/dL)	8.7 mmol/L (157 mg/dL)	8.3 mmol/L (150 mg/dL)	7.5 mmol/L (135 mg/dL)	8.2 mmol/L (148 mg/dL)	6.4 mmol/L (115 mg/dL)	6.9 mmol/L (124 mg/dL)
**Daily nadir bicarbonate level (reference range: 22-32 mmol/L [22-32 mEq/L])**	10 mmol/L (10 mEq/L)	8 mmol/L (8 mEq/L)	9 mmol/L (9 mEq/L)	10 mmol/L (10 mEq/L)	11 mmol/L (11 mEq/L)	10 mmol/L (10 mEq/L)	14 mmol/L (14 mEq/L)	18 mmol/L (18 mEq/L)	24 mmol/L (24 mEq/L)	24 mmol/L (24 mEq/L)	24 mmol/L (24 mEq/L)	25 mmol/L (25 mEq/L)
**Daily peak capillary ketone level (reference range: < 0.6 mmol/L [< 3.49 mg/dL])**	6 mmol/L (35 mg/dL)	4.4 mmol/L (26 mg/dL)	3.8 mmol/L (22 mg/dL)	3.2 mmol/L (19 mg/dL)	1.7 mmol/L (10 mg/dL)	1 mmol/L (5.8 mg/dL)	0.2 mmol/L (1.2 mg/dL)	0.5 mmol/L (2.9 mg/dL)	N/A	N/A	N/A	N/A
**Daily peak lactate level (reference range: 0.5-2.2 mmol/L [4.5-19.8 mg/dL])**	3.2 mmol/L (29 mg/dL)	3.1 mmol/L (28 mg/dL)	4.1 mmol/L (37 mg/dL)	3.5 mmol/L (31.5 mg/dL)	5.0 mmol/L (45 mg/dL)	5.4 mmol/L (48.6 mg/dL)	4.5 mmol/L (41 mg/dL)	3.8 mmol/L (34 mg/dL)	1.9 mmol/L (17 mg/dL)	2.2 mmol/L (20 mg/dL)	2.6 mmol/L (23 mg/dL)	N/A
**Daily peak anion gap (reference range 4-13 mmol/L [4-13 mEq/L])**	16 mmol/L (16 mEq/L)	14 mmol/L (14 mEq/L)	15 mmol/L (15 mEq/L)	14 mmol/L (14 mEq/L)	14 mmol/L (14 mEq/L)	14 mmol/L (14 mEq/L)	10 mmol/L (10 mEq/L)	6 mmol/L (6 mEq/L)	6 mmol/L (6 mEq/L)	9 mmol/L (6 mEq/L)	10 mmol/L (6 mEq/L)	10 mmol/L (6 mEq/L)

Values given are in International System of Units (SI). Values in parenthesis are conventional units. Abbreviation: N/A, data not available.

**Figure 2.**
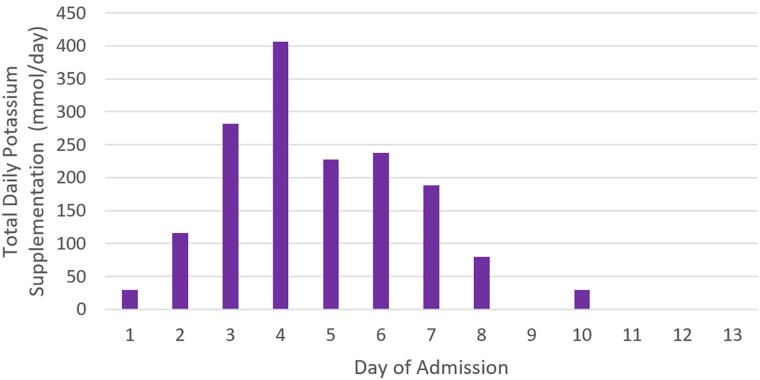
Total daily potassium supplementation requirement (sum of oral and intravenous potassium supplementation for each day during admission). Day 1 of admission was 7 days after the third dose of enfortumab vedotin.

She also experienced significant electrolyte deficiencies, with profound potassium supplementation requirements to a peak of 406 mmol/day on day 4 (Fig. 2) as well as requiring regular intravenous phosphate and magnesium replacement. Her glucose and ketone levels that had previously been only minimally responsive to high insulin doses rapidly improved at day 7 of admission with insulin infusion rates dropping from 50 units/hour to complete cessation within 10 hours. After having insulin glargine the evening prior, intravenous glucose was provided throughout day 7 to prevent hypoglycemia until clearance of all exogenous insulin.

Despite the considerable monitoring and nursing requirements, this patient was able to be successfully managed on the medical/oncology ward, relying on a high level of coordination between members of the endocrine and oncology teams, after-hours medical team, and nursing staff. It also required equipment normally reserved for the intensive care unit to allow intravenous insulin infusion rates well above the 15 units/hour guardrail limitation placed on ward-based insulin infusion pumps.

## Outcome and Follow-Up

Subsequent glucose and ketone monitoring was normal from day 8 of admission onwards. There was a 2-day lag in the resolution of her metabolic acidosis after resolution of her ketosis, likely driven by resolving lactic acidosis. She was discharged home on day 13 on no hypoglycemic agents nor potassium supplementation. She was deemed unsafe to have any further enfortumab vedotin therapy, and subsequently commenced alternate palliative chemotherapy on disease progression 3 months later. Her HbA1c 8 months later was 5.1% (32.2 mmol/mol) (normal reference range: < 5.7%; < 39 mmol/mol).

## Discussion

Since the recent introduction of enfortumab vedotin for the treatment of metastatic urothelial cancer, there have been several reports of enfortumab vedotin–associated diabetic ketoacidosis. A literature review of several databases including PubMed, Embase, and Google Scholar was performed, which revealed 8 published case reports to date (Table 3).

**Table 3.** Summary of case reports for diabetic ketoacidosis associated with enfortumab vedotin and brentuximab vedotin

	Reference	Age, years	Sex	Malignancy	Pre-existing diabetes	Obesity status	Timing of DKA	Biochemistry	Organ failure	ICU admission	Maximum insulin dose (units/day)	HbA1c during admission (reference range: < 5.7% (39 mmol/mol)	HbA1c After discharge, off antidiabetic therapies (reference range: < 5.7% (39 mmol/lmol)	C-peptide	Antibodies	Outcome
**Enfortumab vedotin cases**	[8]	50	M	Metastatic urothelial carcinoma	No	Yes. BMI 35 kg/m^2^	6 days after 2nd dose of EV	pH 7.22, HCO3 8 mmol/L (8 mEq/L) on VBG	AKI, intubated, CRRT	Yes	N/A	7.7% (61 mmol/mol)	N/A	1.67 nmol/L (5.05 ng/mL)	Negative anti-GAD65	Died day 2 of admission.
	[9] [14]	59 59	F F	Recurrent metastatic urothelial carcinoma	Prediabetes	Class 3 obesity	After 2nd dose 1 week after 2nd dose	N/A pH 7.38, ketones 1.4 mmol/L, glucose 26.9 mmol/L (484 mg/dL), HCO3 20 mmol/L (20 mEq/L), lactate 3.5 mmol/L (31.5 mg/dL)	No	Yes	2940	N/A	N/A	14.06 nmol/L (42.48 ng/mL)	Negative anti-GAD65 and IA2	Survived. Transitioned from IV insulin infusion to subcutaneous insulin on day 7. Insulin ceased completely by day 16. Discharged day 18.
	[10]	75	M	Metastatic urothelial carcinoma	N/A	Yes	3 days after 2nd dose	N/A	Yes CRRT	Yes	> 1000	5.3% (34 mmol/mol)	N/A	N/A	Negative anti-GAD and IA2	Died day 3 of admission.
	[11]	57	M	Metastatic urothelial carcinoma	N/A	N/A	6 days after 2nd dose	Glucose >27.7 mmol/L (>499 mg/dL), AG 18 mmol/L (18 mEq/L), positive ketones	Yes CRRT, intubated, refractory shock	Yes	7200	7.7% (61 mmol/mol)	N/A	1.65 nmol/L (4.98 ng/mL)	N/A	Died day 2 of admission.
	[12]	68	M	Advanced urothelial carcinoma	Prediabetes	Class 3 obesity	N/A	N/A	Yes	N/A	900	7.3% (56 mmol/mol)	N/A	12.54 nmol/L (37.9 ng/mL)	Negative insulin antibody, negative anti-GAD, and anti-IA2	Died day 7 of admission.
	[13]	72	M	Metastatic urothelial carcinoma	No	Yes	5 days after second infusion	N/A	Yes. CRRT	Yes	1920	5.3% (34 mmol/mol)	N/A	Elevated (no value provided)	N/A	Died 15 days after presentation.
	[15]	71	M	Invasive high grade papillary urothelial carcinoma	No	Yes. BMI 32 kg/m^2^	1 day after third dose	AG 17 mmol/L (17 mEq/L). Elevated ketones	AKI	Yes	800-1000	7.4% (57 mmol/mol)	5.4% (35.5 mmol/mol)	6.17 nmol/L (18.64 ng/mL)	Negative anti-GAD, IA2, ZnT8, insulin antibodies	Survived. Discharged on glipizide. By one year later was weaned off all hypoglycemic agents with normal HbA1c 5.4%. EV discontinued.
	[16]	71	M	Recurrent metastatic urothelial carcinoma with lung metastases	Prediabetes	N/A	5 days after third dose	pH 7.29, ketosis	Yes CRRT	Yes	2160	6.1% (43 mmol/mol)	N/A	> 9.9 nmol/L (>29.9 ng/mL)	Negative anti-GAD antibody	Died. Transitioned from IV to subcutaneous insulin by day 9, then developed organizing pneumonia and diet day 21 of admission.
	**Current case Report**	**62**	**F**	**Metastatic urothelial carcinoma**	**No**	**BMI 25.6 kg/m^2^**	**7 days after 3rd dose of EV**	**Ketones 6.0 mmol/L, pH 7.21, HCO3 10 mmol/L (10 mEq/L), AG 16 mmol/L (16 mEq/L)**	**No**	**No**	**1213**	**6.7% (50 mmol/mol)**	**5.1% (32.2 mmol/mol)**	**6.3 nmol/L (19.03 ng/mL)**	**Negative GAD/IA2 antibodies, negative insulin antibodies**	**Survived. Complete resolution of ketosis and hyperglycemia by day 7 of admission, discharged on day 13 with normal BGLs and no hypoglycemic agents.**
**Brentuximab vedotin cases**	[17]	23	M	Hodgkin lymphoma	No	Yes BMI 42.7 kg/m^2^	1 week after most recent BV dose during 2nd cycle	Glucose 16.9 mmol/L (304 mg/dL), pH 7.25, HCO3 14.9 mmol/L (14.9 mEq/L), ketones 6.2 mmol/L	No	Unknown	1700	6.8% (51 mmol/mol)		9.24 nmol/L (27.92 ng/mL)	Negative anti-insulin, anti-GAD, and anti-IA2 antibodies	Survived. DKA resolved by day 8 of admission, IV insulin infusion until day 11, then commenced on basal/bolus subcutaneous insulin. Discharged on day 12 with discontinuation of all hypoglycemics at 2 months with HbA1c at 3 months post DKA of 5.3%.
	[18]	54	M	Hodgkin lymphoma	No	Yes. BMI 37 kg/m^2^	1 week after first dose of BV	Glucose 25.0 mmol/L (450 mg/dL), HCO3 7 mmol/L (7 mEq/L), ketones 11.2 mmol/L	Cardiovascular, respiratory, and renal failure with lactate to >28 mmol/L (>252 mg/dL)	Yes	10 725	N/A		5.02 nmol/L (15.17 ng/mL)	GAD and IA2 antibodies negative; negative anti-insulin receptor antibodies	Died from multi-organ failure 72 hours after admission.
	[19]	47	F	Hodgkin lymphoma	No	BMI 26.96 kg/m^2^	2 weeks after first dose of BV	Glucose 23.8 mmol/L (428 mg/dL), AG 18 mmol/L, pH 7.32, HCO3 16.1 mmol/L (16.1 mEq/L), serum ketones 4.3 mmol/L	No	Yes	1080	6.7% (50 mmol/mol)		N/A	N/A	Survived. Weaned off IV insulin infusion by day 6 of admission but remained on subcutaneous insulin until day 8. Discharged on day 10 with no insulin or hypoglycemic medication. No further BV doses.

Current case report is shown in bold font. Values in parenthesis are in conventional units. Abbreviations: AG, anion gap; AKI, acute kidney injury; BMI, body mass index; BV, brentuximab vedotin; CRRT, continuous renal replacement therapy; DKA, diabetic ketoacidosis; EV, enfortumab vedotin; GAD, glutamic acid decarboxylase; HCO3, bicarbonate; IA2, islet antigen 2; ICU, intensive care unit; IV, intravenous; N/A, not available; VBG, venous blood gas; ZnT8, zinc transporter 8.

The consistent mechanism driving enfortumab vedotin–associated DKA across all these case reports was severe insulin resistance as evidenced by hyperglycemia with elevated C-peptide levels, negative diabetes antibodies, and massive insulin dose requirements, with peak doses ranging from 800 to 7200 units per day. For patients that survived to discharge, this insulin resistance was transient and fully reversible, requiring minimal to no hypoglycemic agents at discharge. The timing for the onset of enfortumab vedotin–related DKA varies significantly but has been reported to occur from 3 days after the second dose.

The mechanism of how enfortumab vedotin causes insulin resistance and DKA is not well understood. To explore this further, we performed a literature review of other ADCs that utilize the same cleavable linker and payload. Three other ADCs were identified: brentuximab vedotin, polatuzumab vedotin, and pinatuzumab vedotin. (Table 3) As with enfortumab vedotin, the 3 cases of brentuximab vedotin–associated DKA were associated with severe insulin resistance, with insulin requirements up to 10 725 units per day in one case [18]. Despite both polatuzumab vedotin and pinatuzumab vedotin being associated with hyperglycemia, there has yet to be reported cases of DKA secondary to these agents [20].

As outlined in Table 3, most patients who developed enfortumab vedotin– or brentuximab vedotin–associated DKA did not have known pre-existing diabetes. Although it is possible that pre-existing diabetes were underdiagnosed, most patients would be screened for diabetes given findings from the EV-301, where prior HbA1c ≥ 7.0% increases the risk of hyperglycemia [6]. Interestingly, some patients without pre-existing diabetes, like our patient, had elevated HbA1c levels at time of admission followed by later normalization of HbA1c [8, 15]. The exact reason of transiently elevated HbA1c is yet to be understood. Those who experienced multi-organ failure due to refractory DKA did not survive their admission, highlighting the lethal nature of this TRAE. The current mortality rate for all published cases is 67% for enfortumab vedotin–associated DKA (including this case) and 33% for brentuximab vedotin–associated DKA.

Various mechanisms that may lead to off-target effects of enfortumab vedotin have been postulated. Firstly, the relatively unstable cleavable linkers can result in premature deconjugation of MMAE in the plasma leading to passive diffusion into nontarget cells through plasma membranes. The half-life for unconjugated MMAE is 3.0 to 5.1 days and reaches a peak plasma concentration 2 to 4 days after ADC infusion [21]. The enfortumab vedotin product information reports the ADC half-life as 2.6 days and 3.6 days for MMAE [22]. Secondly, the intact ADC can be taken in by nontarget cells via nonspecific endocytosis before then releasing the MMAE payload into these nontarget cells [19]. Finally, there may also be the bystander effect, where the internalized payload is then able to diffuse back into the extracellular space into surrounding cells, irrespective of the expression of surface target antigen, and exert cell killing [23].

One or a combination of these mechanisms may explain the timeline seen in the reported cases. Our patient took 14 days to fully normalize all glucose measurements, which is in keeping with 4- to 5-times the ADC half-life. For the other enfortumab vedotin DKA cases who survived (Table 3), 1 patient presented with mild DKA 7 days after the most recent dose of enfortumab vedotin with full resolution after 16 days [9, 14]. For the brentuximab vedotin cases, 1 patient had resolution of DKA by day 8 of admission with intravenous insulin ceased day 11 [17], while another by day 8 [19].

MMAE is very likely the causative drug with this effect seen across multiple ADCs that share this payload, but it is not fully understood how MMAE results in insulin resistance. The marked potassium requirements in this case support the preservation of insulin action at the cell surface level, acting upon the Na^+^/K^+^ ATPase pump to cause hypokalemia. We would thus expect insulin to also act on cell surface receptor tyrosine kinases activating PI3K/AKT intracellular signaling. However, we postulate that MMAE could directly disrupt the microtubule action responsible for intracellular trafficking of glucose transporter 4 (GLUT4), which occurs downstream of PI3K/AKT activation, resulting in failure of GLUT4 exocytosis, inability for glucose transport across plasma membrane and thus insulin resistance [24]. The presence of ketosis suggests insulin resistance at the level of adipocytes and liver, causing lipolysis, free fatty acid oxidation, and ketogenesis [25].

The management of enfortumab vedotin–associated insulin resistance is challenging. Early recognition of the potential for rapid deterioration is lifesaving. It is imperative that the completely reversible nature with rapid resolution upon full elimination of MMAE is also recognized. Therefore, metastatic disease status should not be a deterrent to receiving supportive treatment in the intensive care setting. These patients may often require intensive clinical monitoring beyond the capacity of standard ward-based care, higher insulin infusion rates, and central venous access for very high electrolyte replacement requirements. However, this case shows that ward-based care is possible through effective collaboration and communication between medical and nursing staff.

This case highlights a rare TRAE of enfortumab vedotin–related DKA, which is life-threatening although reversible in those patients who do not experience multi-organ failure. As we expect a trend toward more enfortumab vedotin use, more studies are required to explore the pathophysiology behind insulin resistance following the use of MMAE, to guide risk stratification, prevention, and treatment of enfortumab vedotin–associated DKA.

## Learning Points

- Enfortumab vedotin–associated DKA is a rare drug adverse event driven by transient but severe insulin resistance and can occur in patients with no prior history of diabetes.
- Enfortumab vedotin–associated DKA has a high mortality rate but if a patient can survive until drug clearance, then enfortumab vedotin–associated diabetes can be fully reversible.
- With rapid insulin resistance reversibility, we would recommend using only intravenous insulin and short acting subcutaneous insulin therapy and avoid longer acting subcutaneous insulin.
- Monomethyl auristatin E (MMAE) is the likely culprit drug as this complication has also been observed in other agents that utilize MMAE as their payload.
- It is important to aggressively replace electrolytes, especially potassium, during enfortumab vedotin–associated DKA.

## Data Availability

Data sharing is not applicable to this article as no datasets were generated or analyzed during the current study.

## Abbreviations

ADC: antibody-drug conjugate
DKA: diabetic ketoacidosis
HbA1c: glycated hemoglobin
MMAE: monomethyl auristatin E
TRAE: treatment-related adverse event
